# Comparison of Mechano- and PhotoATRP with ZnO Nanocrystals

**DOI:** 10.1021/acs.macromol.3c00250

**Published:** 2023-06-26

**Authors:** Martin Cvek, Arman Moini Jazani, Julian Sobieski, Thaiskang Jamatia, Krzysztof Matyjaszewski

**Affiliations:** † Department of Chemistry, Carnegie Mellon University, 4400 Fifth Avenue, Pittsburgh, Pennsylvania 15213, United States; ‡ Centre of Polymer Systems, Tomas Bata University in Zlin, Trida T. Bati 5678, 760 01 Zlin, Czech Republic

## Abstract

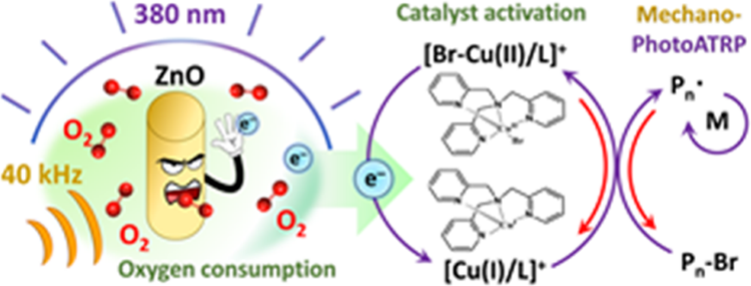

Zinc oxide (ZnO) was previously reported as an excellent cocatalyst for mechanically controlled atom transfer radical polymerization (mechanoATRP), but its photocatalytic properties in photoinduced ATRP (photoATRP) have been much less explored. Herein, well-defined ZnO nanocrystals were prepared via microwave-assisted synthesis and applied as a heterogeneous cocatalyst in mechano- and photoATRP. Both techniques yielded polymers with outstanding control over the molecular weight, but ZnO-cocatalyzed photoATRP was much faster than analogous mechanoATRP (conversion of 91% in 1 h vs 54% in 5 h). The kinetics of photoATRP was tuned by loadings of ZnO nanocrystals. PhotoATRP with ZnO did not require any excess of ligand versus Cu, in contrast to mechanoATRP, requiring an excess of ligand, acting as a reducing agent. ZnO-cocatalyzed photoATRP proceeded controllably without prior deoxygenation, since ZnO was involved in a cascade of reactions, leading to the rapid elimination of oxygen. The versatility and robustness of the technique were demonstrated for various (meth)acrylate monomers with good temporal control and preservation of end-group functionality, illustrated by the formation of tailored block copolymers.

## 1 Introduction

Atom transfer radical polymerization (ATRP) is one of the most efficient synthetic techniques to prepare well-defined polymers with complex architecture.^1−3^ ATRP is mediated by an equilibrium between alkyl halides activated by Cu(I) complexes to reversibly generate propagating radicals and Cu(II) deactivators. Due to unavoidable radical termination, a fraction of Cu(I) activators irreversibly convert to Cu(II) deactivators over the course of the reaction, and ATRP can stop when all activators are consumed. To diminish the concentration of Cu-based ATRP catalysts and reach high monomer conversion, it is necessary to (re)generate Cu(I)/L activators from Cu(II)/L deactivator species by various chemical reducing agents (e.g., ascorbic acid, tin(II) ethylhexanoate), radical initiators such as azobisisobutyronitrile (AIBN),^4^ or using external stimuli,^5−7^ including electrical current,^8,9^ light in the presence of external electron donors,^10,11^ or mechanical energy.^12^ These approaches have gained significant attention, as they allow the on-demand spatiotemporal control over the reaction kinetics,^13^ under mild conditions.^5^

In mechanically controlled ATRP (mechanoATRP), the electrons are generated by piezoelectric nanoparticles dispersed in the ATRP solution upon exposure to ultrasonic shock waves or impact forces, such as ball milling,^14^ thereby reducing the Cu(II) catalyst deactivator to form Cu(I) activators. The extent of the electron generation can be controlled by loading of the piezoelectric agents^15^ or tuning their physical properties (i.e., dimensions, crystallographic structure, etc.).^16,17^ The selection of piezo-active agents is limited mostly to semiconductors, such as barium titanate (BaTiO_3_) and zinc oxide (ZnO) nanoparticles. Indeed, ZnO exhibited a superior efficiency to BaTiO_3_ in the mechanoATRP of acrylates due to its lower band gap. Whereas the effective loading of ZnO nanoparticles ranged from 0.6 to 1.2 wt % (relative to the monomer and solvent),^17^ the BaTiO_3_ counterparts typically required loadings in the range of 0.9–9.0 wt %.^15^

Photoinduced ATRP (photoATRP) employs the photochemical reaction of excited Cu(II)/L deactivators with electron donors, such as amines (including also excess N-based ligands).^18,19^ Alternatively, organic dyes, such as eosin Y,^20^ upon excitation can form strongly reducing species that can directly reduce Cu(II) deactivators by outer sphere electron transfer (OSET) in the oxidative quenching process. Also, the excited photocatalysts can first react with electron donors and form radical anion species, which can transfer an electron and reduce Cu(II) species to Cu(I) activators. The organic dyes may cause coloration of the polymer product and their extraction is challenging.^21^ In some cases, they may experience photobleaching and a consequential loss of photocatalytic efficiency.^20^ Such drawbacks can be avoided by employing heterogeneous photocatalysts, allowing their easier removal and higher optical stability.^22,23^ Some heterogeneous photocatalysts may even provide further benefits, e.g., silica-coated Fe_3_O_4_ nanoparticles with immobilized rhodamine B were magnetically separated and reused.^24^ In addition, lanthanide-doped upconversion nanoparticles (UCNPs), activated by near-infrared (NIR) light, deeply penetrated various reaction vessels.^25^

Zinc oxide is a remarkable semiconductor with favorable piezoelectric^16,26^ and photocatalytic properties, which render this material an attractive candidate for heterogeneous catalysis.^27,28^ In polymer synthesis, the piezo/photocatalytic properties of ZnO were studied primarily as monocatalytic systems. The ZnO photocatalysts were previously used to photoinitiate free radical polymerization of *N*,*N*-dimethylacrylamide hydrogels when exposed to sunlight irradiation.^29^ ZnO was also employed in photoATRP^30^ and photoinduced electron transfer–reversible addition–fragmentation chain transfer (PET-RAFT) of methyl methacrylate (MMA).^31^ Recently, it was applied in piezoelectrically mediated RAFT to cure composite resins.^32^

Nevertheless, all these ZnO-cocatalyzed reversible deactivation radical polymerization (RDRP) techniques reported high oxygen sensitivity and required deoxygenation procedures, either by freeze–pump–thaw (FPT) cycles^16,26,30,32^ or by inert gas purging.^17,31^ Often, low monomer conversions were achieved after relatively long irradiation times (several hours).^31^ In ATRP, a several-fold excess of expensive ligands was required.^30^ To meet current economic and environmental requirements, it is important to diminish the concentration of ligands and avoid additional unnecessary procedures such as deoxygenation.^33,34^

**Scheme 1:**
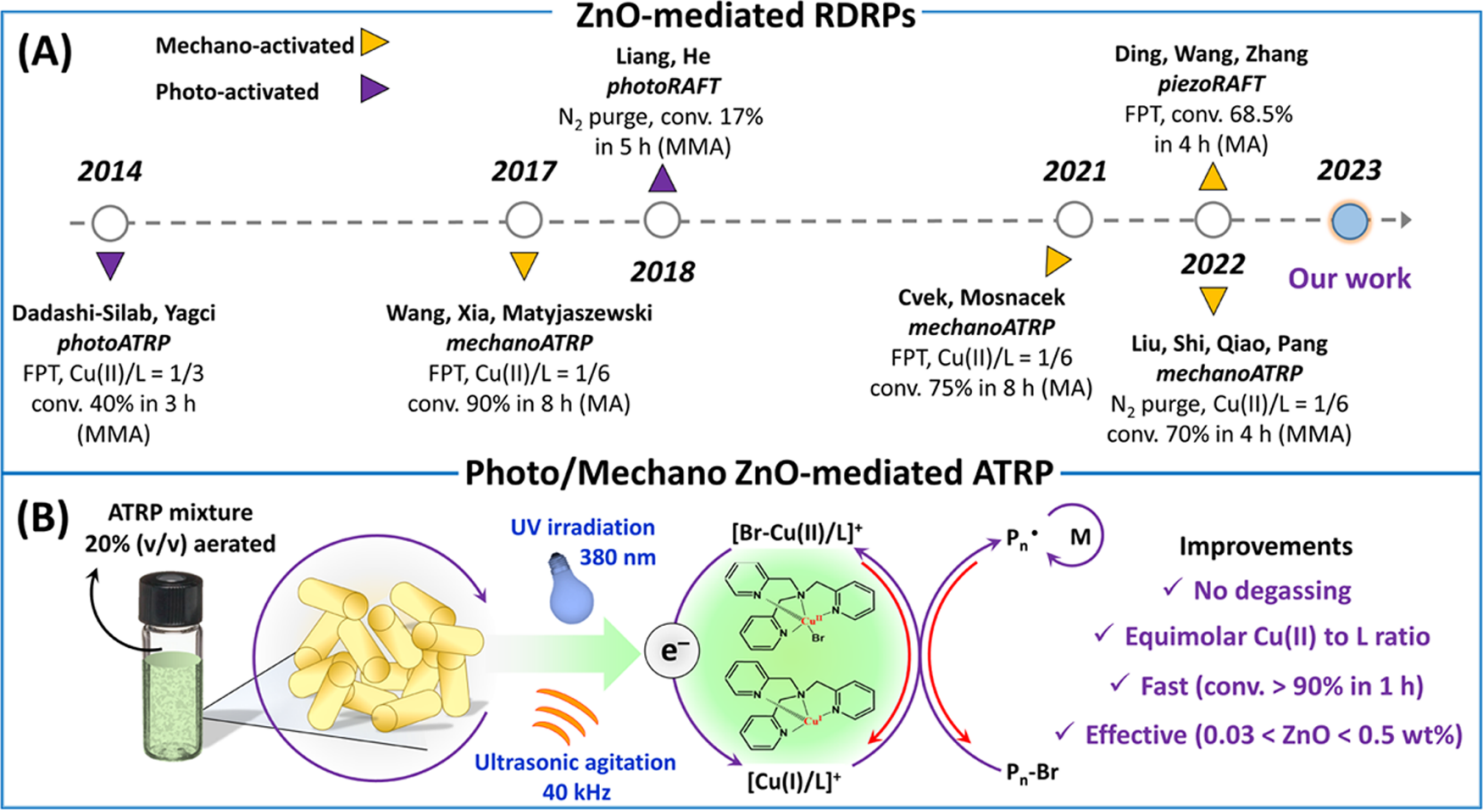
(A) Development of the ZnO-Mediated RDRPs. (B) Representation of the Proposed Methodology with the Suggested Improvements

Herein, the performance of well-defined ZnO nanocrystals as cocatalysts in both photo- and mechanoATRP is investigated. Scheme 1A presents historical developments in ZnO-cocatalyzed RDRPs. Scheme 1B illustrates currently studied systems, with highlighted improvements based on avoiding a degassing process using stoichiometric concentrations of Cu and the ligand, decreasing the amount of ZnO down to 0.03 wt % and accelerating polymerization of (meth)acrylates to reach 90% conversion in 1 h. Well-defined polymers under temporal control were prepared with preservation of chain functionality. ZnO-cocatalyzed mechano/photoATRP techniques can be synergistically combined, expanding the scope of the applicability of this dualistic catalytic system.

## 2 Results and Discussion

### 2.1 Synthesis and Characterization of ZnO Nanocrystals

**Figure 1:**
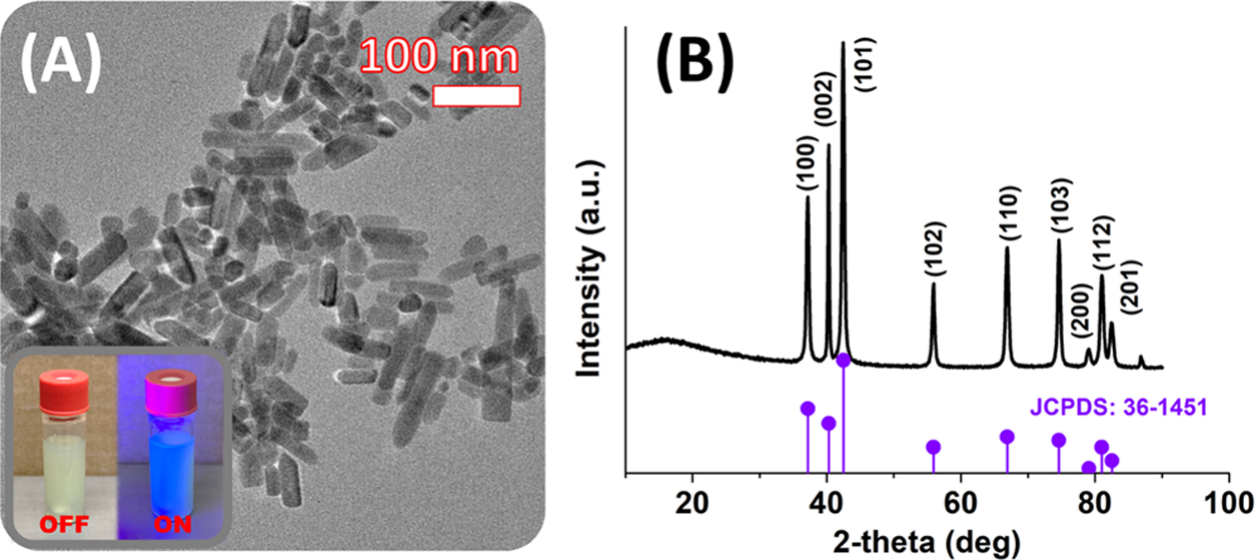
(A) TEM image of ZnO nanocrystals (inset, digital photo of reaction dispersion before and after UV light irradiation). (B) XRD pattern for the ZnO nanocrystals, with vertical lines representing the Bragg positions for the corresponding JCPDS.

ZnO nanocrystals were prepared through the microwave (MW)-assisted polyol-mediated synthesis^35^ and applied as an effective cocatalyst in the externally controlled ATRPs of various (meth)acrylate monomers. To prevent aggregation and ensure the stability of the ATRP dispersion, the surface of the ZnO nanocrystals was treated with oleic acid (OA; Section 1.2. in the Supporting Information (SI)). This surfactant was selected because it avoids the problem of nitrogen-containing compounds, such as hexamethylenetetramine, octylamine, or triethanolamine^27^ that could act as reducing agents and circumvent the ZnO-cocatalyzed mechano- and photoATRP process or potentially ligate to Cu centers. As shown by TEM (Figure 1A), the ZnO particles possessed an oblate/rod-like shape with dimensions of around 56 ± 19 nm × 20 ± 3 nm (aspect ratio of ∼2.8), as determined by the image analysis. The crystallographic structure was analyzed using the XRD technique (Figure 1B), showing the diffraction peaks at 2θ of 37.2, 40.3, 42.5, 55.9, 66.9, 74.6, 79.1, 81.0, and 82.5°, which were attributed to (100), (002), (101), (102), (110), (103), (200), (112), and (201) crystallographic planes, respectively. Such a pattern corresponded to the hexagonal wurtzite structure,^17^ which is in good agreement with standard JCPDS 36-1451. The high intensity of sharp diffraction lines confirmed the formation of a highly crystalline material. The average crystallite size, *D*, was calculated according to the Scherrer equation that reads as follows
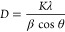
where *K* is Scherrer’s constant (a typical value is 0.9), λ is the wavelength of the X-ray source, λ(CoKα_1,2_) = 1.7903 Å, β is the full width at half-maximum (FWHM) intensity of a peak observed at a mean scattering 2θ angle (expressed in radians), and θ is the Bragg angle.^36^ The calculated *D* was 25.3 nm, which is a very close value to the size of the individual nanocrystals. Optical properties, such as absorbance and the band gap energy, of the powdered ZnO were investigated using DRUV–vis spectroscopy. The results showed predominant absorption in the UV region with a sharp absorbance cutoff at wavelengths greater than 380 nm (Figure S1A), which directed the wavelength selection of our excitation source for the photoATRP experiments. Further, the band gap energy value determined using Tauc’s method^37^ was ∼3.24 eV (Figure S1B).

### 2.2 Optimization of ZnO-Cocatalyzed ATRPs

**Figure 2:**
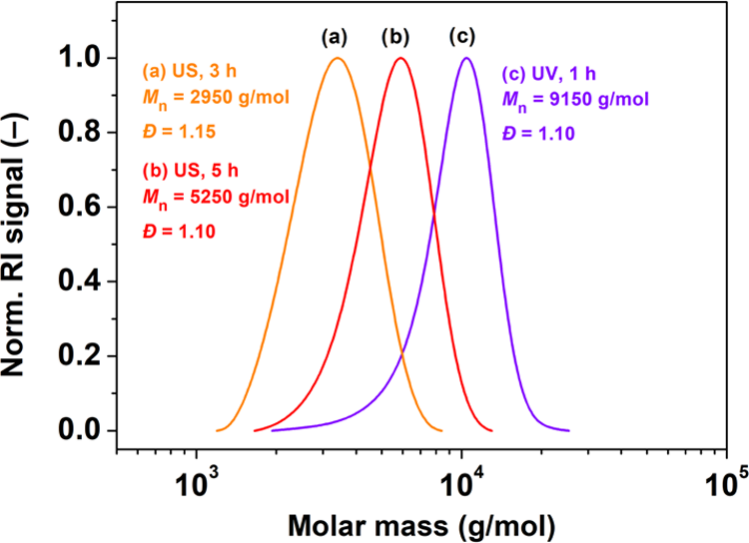
Direct comparison of the PMA prepared by mechano- and photoATRP cocatalyzed by ZnO nanocrystals (0.5 wt %). Reaction conditions: [MA]_0_/[EBiB]_0_/[CuBr_2_]_0_/[TPMA]_0_ = 100/1/0.04/0.16 in 50% (v/v) DMSO without prior deoxygenation, closed-cap reactor bearing 20% (v/v) of aerated headspace, ultrasound source (40 kHz, 110 W), and UV light source (380 nm, 28.5 mW/cm^2^). Synthesized PMA: (a) after 3 h of ultrasonic agitation, (b) after 5 h of ultrasonic agitation, and (c) after 1 h of UV irradiation.

We initially studied the effect of the MW-synthesized ZnO nanocrystals (0.5 wt %) on the polymerization efficiency of methyl acrylate (MA) using dimethyl sulfoxide (DMSO) as a solvent, copper(II) bromide/tris(2-pyridylmethyl)amine (CuBr_2_/TPMA) as a catalytic system, and ethyl-α-bromoisobutyrate (EBiB) as the ATRP initiator.^16,17,26^ The ATRP mixture was not deoxygenated, and the aerated headspace of the closed reactor represented approx. 20% (v/v). When using a [CuBr_2_]/[TPMA] ratio of 1/6 as reported previously in the literature,^16,17,26^ ZnO-cocatalyzed photoATRP (380 nm, 28.5 mW/cm^2^) resulted in a gel-like product, which was attributed to excessively generated activating species, leading to poor control over polymerization. Therefore, the concentration of the ligand decreased (CuBr_2_/L loading ratio of 1/4), which resulted in a high conversion of the monomer (91%) in a relatively short time and a gained control (*Đ* = 1.10) over the reaction (Table 1, entry 1). The analogous mechanoATRP with ultrasound sonication (40 kHz, 110 W) was also controlled but proceeded at a much slower rate, providing moderate conversion, e.g., 54% in 5 h (Table 1, entry 2). This suggested a superior photocatalytic activity of the ZnO nanocrystals in ATRP compared to their mechanically stimulated ability to generate the radicals, as the rate-determining step of regenerative ATRP techniques is reduction. Furthermore, the GPC traces were monomodal without tailing or high-molecular-weight (HMW) shoulder for both Zn-cocatalyzed photo- and mechanoATRP (Figure 2).^26^

**Table 1:** Assessment of ZnO Nanocrystals as Heterogeneous Catalysts for Mechano- and PhotoATRP of Methyl Acrylate

entry^a^	ZnO (wt %)	stimulus	CuBr_2_ (equiv.)	TPMA (equiv.)	time (h)	conv.^b^ (%)	*M*_*n*,th_^c^	*M*_*n*,GPC_^d^	*Đ*^d^ (−)
1	0.5	UV	0.04	0.16	1	91	8030	9150	1.10
2	0.5	ultrasound	0.04	0.16	1	0			
					3	30	3040	2950	1.15
					5	54	4840	5250	1.10
3	0.5	UV	0.04	0.08	1	90	7940	7470	1.10
4	0.5	ultrasound	0.04	0.08	1	0			
					3	<5		<500	
					5	16	1570	2250	1.14
5	0.5	UV	0.04	0.04	1	85	7510	8030	1.11
6	0.5 or 1.0	ultrasound	0.04	0.04	5	0			

a Reaction conditions: [MA]_0_/[EBiB]_0_/[CuBr_2_]_0_/[TPMA]_0_ = 100/1/0.04/0.04–0.16 in 50% (v/v) DMSO without prior deoxygenation, closed-cap reactor bearing 20% (v/v) of aerated headspace; ultrasound source (40 kHz, 110 W), UV light source (380 nm, 28.5 mW/cm^2^), ambient temperature.

b Determined from ^1^H NMR spectra (CDCl_3_ as the solvent).

c Calculated following the equation *M*_*n*,th_ = *M*_EBiB_ + [MA]_0_/[EBiB]_0_ × conversion × *M*_MA_.

d Determined by GPC analysis (THF as the eluent) calibrated to a linear PMMA standard.

After decreasing the ratio of CuBr_2_ and the ligand to 1/2, the performance of ZnO-cocatalyzed photoATRP changed rather marginally (Table 1, entry 3), while the analogous mechanoATRP significantly slowed down (Table 1, entry 4). The next experiments were conducted using equimolar loadings of CuBr_2_ and the ligand, on which regenerative ATRP techniques typically rely to act as electron donors. For example, equimolar loadings of CuBr_2_ and the ligand in previously reported studies yielded high *Đ* values, as in the oxygen-tolerant photoATRP of *N*-isopropylacrylamide (NIPAM)^38^ or negligible monomer conversion in photoATRP of MA catalyzed by conjugated microporous polymers.^21^ Surprisingly, ZnO-cocatalyzed photoATRP under such uncommon conditions (Table 1, entry 5) provided a high monomer conversion of 85% with a high degree of control (*Đ* = 1.11) in a relatively short time (1 h), suggesting that the ZnO nanocrystals acted as efficient reducing agents in the absence of excess ligand. This result also indicates that classical photoATRP with regeneration by amines is not the dominant catalyst regeneration pathway in the ZnO photoATRP system.

On the contrary, no monomer conversion was observed in the analogous mechanoATRP, even after increasing the ZnO loading (Table 1, entry 6). It is known that the ZnO catalyst in mechanoATRP requires electron donation from excess amines to efficiently replenish electron holes.^26^ This could be caused by a less efficient transfer and/or generation of electron–hole pairs under mechanical stimulation (under herein applied intensity) of ZnO compared to UV photoirradiation. The control mechanoATRP, under equimolar CuBr_2_/L conditions, in the absence of ZnO nanocrystals was not successful. In photoATRP without ZnO, the formation of new initiating chains from radical cations originating from excess ligand (electron donor) was diminished by decreasing the ligand loading, but the equimolar loading of CuBr_2_ to the ligand resulted in no monomer conversion (Table S1).

### 2.3 Effects of Other Factors in ZnO-Cocatalyzed PhotoATRP

To clarify the role of ZnO in photoATRP, a series of control experiments were performed. The experiment without the CuBr_2_/L catalytic system resulted in the formation of a gel-like product, indicating that ZnO nanocrystals can participate in the generation of radicals, initiating free radical polymerization (FRP; Table S2, entry 1).^22^ After removing the ATRP initiator, EBiB, a small conversion was detected, suggesting the coinitiation role of the UV-irradiated ZnO (Table S2, entry 2). The molecular weight of the as-prepared product was relatively high, demonstrating a low number of polymer chains generated and, hence, low initiation efficiency. In contrast, the complete ATRP system provided higher conversion and molecular weight of PMA, comparable with the theoretical values (Table S2, entry 3). To conclude, ZnO has a minor coinitiation role compared to its cocatalyst role in photoATRP.

Additionally, the effect of CuBr_2_ on the ZnO-cocatalyzed photoATRP of MA was investigated, under equimolar CuBr_2_/L conditions. When the concentration of CuBr_2_/L was decreased by half while keeping the same ZnO loading, the reaction proceeded faster (Table S3, entry 1 vs 2, and entry 3 vs 4), since a higher percentage of the deactivator species was converted to activators, which brought up a lower reaction control and higher *Đ* values. For this reason, the ZnO loading decreased simultaneously with the CuBr_2_/L concentration (Table S3, entry 2 vs 3, and entry 4 vs 5). The results show that it was possible to systematically suppress the CuBr_2_ and ligand concentrations down to 100 ppm while keeping ZnO-cocatalyzed photoATRP highly effective. However, due to the difficult handling of extremely low ZnO loadings and a lower control over photoATRP, we selected the CuBr_2_ and TPMA concentrations of 400 ppm for the subsequent experiments, as it suggested the best matching between the reduction and propagation rates.

### 2.4 Monitoring of Oxygen Concentration

**Figure 3:**
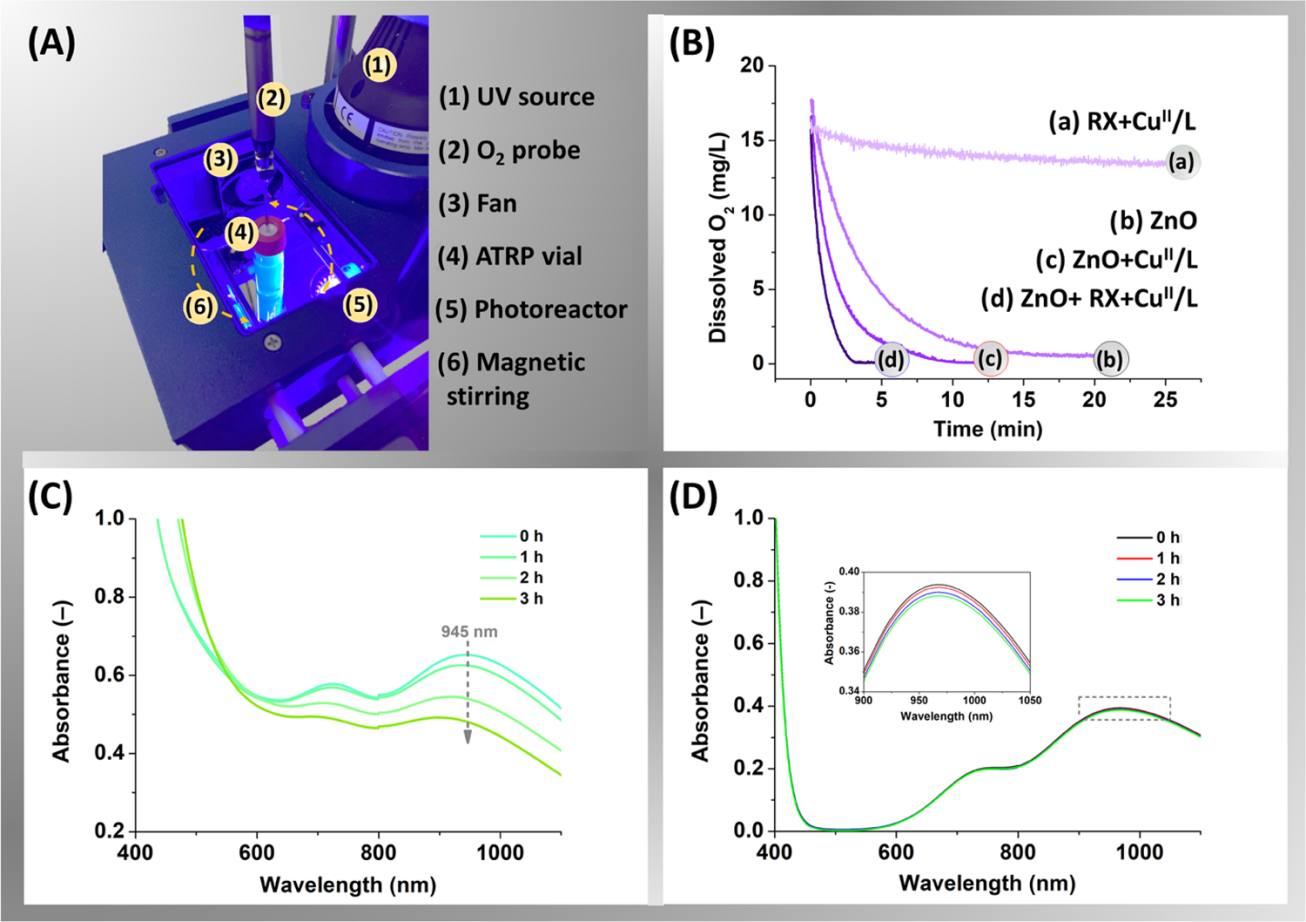
(A) Experimental setup for the oxygen concentration measurements, (B) the effects of the ZnO nanocrystals (0.25 wt %) and the other ATRP components (excluding monomer) on the consumption of dissolved oxygen upon UV light irradiation (380 nm, 28.5 mW/cm^2^, closed-cap reactor bearing 20% (v/v) of aerated headspace), reaction conditions: [EBiB]_0_/[CuBr_2_]_0_/[TPMA]_0_ = 1/0.04/0.04 in DMSO, (C) vis–NIR spectra of [CuBr_2_]_0_/[TPMA]_0_ = 0.04/0.04 complexes in DMSO with ZnO nanocrystals (0.06125 wt %), and (D) without the ZnO nanocrystals at different irradiation times.

ATRP typically requires deoxygenation of the reaction mixture, as oxygen can scavenge radicals and form inactive peroxy radicals,^38−40^ as well as render Cu(I) activator species inert through trapping and formation of oxygen-bearing Cu(II) species. Since ZnO-cocatalyzed photoATRP reactions proceeded without applying a deoxygenation procedure,^41^ the effects of ZnO nanocrystals and the other reaction components (excluding monomer) on the oxygen levels in the ATRP reactor were examined. In each experiment (Figure 3A), the oxygen probe was inserted into a sealed vessel through a septum, and upon UV irradiation (380 nm, 28.5 mW/cm^2^), the concentration of the dissolved oxygen was measured as a function of time. As shown in Figure 3B, in the system with only ZnO (0.25 wt %) dispersed in DMSO, the level of dissolved oxygen steeply decreased and was almost fully depleted (≤0.6 mg/L) within ∼15 min of irradiation. This phenomenon was attributed to the heterogeneous photocatalytic oxidation, in which the photonic energy generates an electron–hole pair in ZnO, which is involved in the redox reactions. In particular, the electrons react with oxygen molecules to produce superoxide radical anions and subsequently hydroxyl radicals, which can oxidize DMSO into dimethyl sulfone (DMSO_2_).^27,42^ To support this reaction pathway, an increasing concentration of DMSO_2_, as the end product, was detected by ^1^H NMR spectroscopy during UV irradiation. Apparently, the amount of oxygen in the closed-cap reactor was not enough to generate a detectable amount of DMSO_2_; thus, the reaction was further continued under open-air conditions. This resulted in a clear increase of the DMSO_2_ peak (3.0 ppm) in the ^1^H NMR spectrum over time (Figure S2).

In the presence of CuBr_2_/TPMA, oxygen was consumed faster, within ∼9 min of photoirradiation. Such acceleration was attributed to the rapid reaction of CuBr/TPMA (formed by reduction of CuBr_2_/TPMA with ZnO) with oxygen. Even faster elimination of oxygen (≤0.1 mg/L), within ∼3 min, was achieved in the presence of an initiator, EBiB, and CuBr_2_/TPMA. This was associated with the rapid reaction of radicals with oxygen, as reported before.^40^ In a control experiment, without ZnO, the UV irradiation yielded a minimal effect on oxygen levels. Overall, the presence of ZnO was the dominating factor responsible for oxygen scavenging, and thus, ZnO-cocatalyzed photoATRP was feasible without applying prior deoxygenation, even in reactors bearing 20% (v/v) of aerated headspace.

### 2.5 Photoreduction of Cu Complexes and Proposed Mechanisms

Additionally, vis–NIR spectroscopy was used to monitor the effect of ZnO on the photoreduction of Cu complexes, in the absence of MA and EBiB (without deoxygenation, Figure 3C). The absorbance band related to CuBr_2_/TPMA gradually decreased with irradiation time, demonstrating the conversion of Cu(II)/L deactivator species into Cu(I)/L activator species, which bear negligible absorption in the near-IR region.^42^ This process was accompanied by a color change from light green to yellowish, though the latter may be due to oxidized entities that accumulate in the reaction and not to the Cu(I)/L catalyst itself, which should be rather colorless. On the contrary, no photoreduction was observed in the absence of ZnO nanocrystals (Figure 3D), and the results imply that ZnO effectively generated Cu(I)/L activators photochemically.

**Scheme 2:**
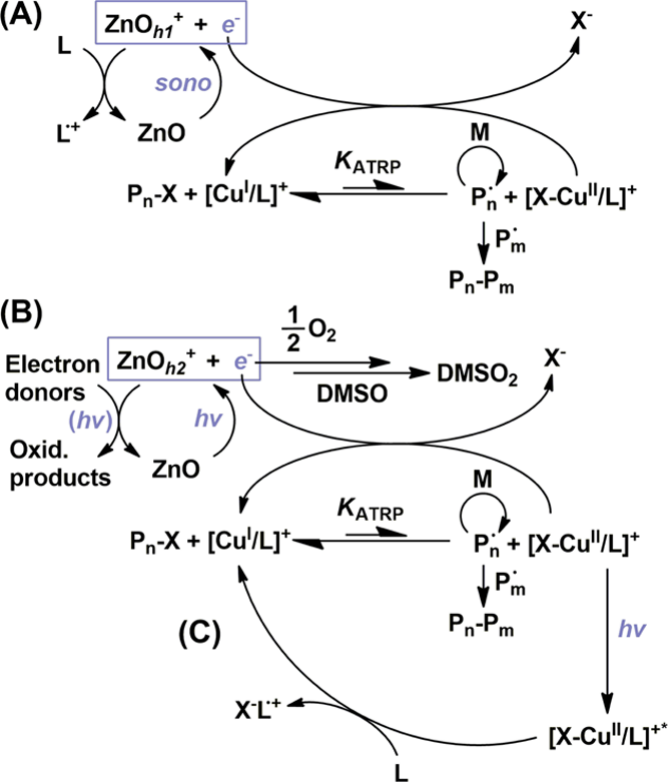
Proposed Dominant Mechanisms for ZnO-(Co)catalyzed (A) Mechano- and (B) PhotoATRP Systems, Where (C) Designates Classical PhotoATRP and Proceeds via the Photoexcited Deactivator Complex [X–Cu^II^/L]^+^* Pathway (B) includes a cascaded reaction for the oxidation of DMSO catalyzed by ZnO and light, as well as potentially various eligible electron donors to form oxidized products (oxid. products), in which light may be further implicated as designated by *hv*. The species ZnO_*h*1_^+^ and ZnO_*h*2_^+^ indicate corresponding electron holes made by mechano- and photostimuli methods, respectively. Disproportionation termination products, organometallic, and oxygen-bearing Cu(II) species are omitted.

The major processes of reducing [Br–Cu(II)/L]^+^ deactivator species to [Cu(I)/L]^+^ activator species share resemblance between the mechano- and photostimulated ZnO-(co)catalyzed pathways, but subtle differences and implications exist (Scheme 2). Both pathways are dominated by the excitation and donation of an *e*^–^ from ZnO to ground-state [Br–Cu(II)/L]^+^ and, consequently, the formation of an electron hole (*h*^+^), which must be regenerated by an electron donor. However, as suggested by Table 1, entries 2–5, the mechanoATRP pathway required excess amine/ligand loading to serve this function and drive successful ATRP, while the analogous photoATRP did not. This could be attributed to different quenching capabilities. MechanoATRP proceeded strictly through the well-documented use of TPMA (amine) as an electron donor.^26^ On the other hand, the photoATRP pathway appeared to proceed through other electron donors in the system, potentially further accessed by photoirradiation that could allow otherwise inert electron donors to perform electron transfer (see Supporting Information, Section 2.1, for further discussion).

The photoATRP pathway can innately possess a classical photoATRP activator regeneration process due to the presence of [Br–Cu(II)/L]^+^ deactivators, which can photoexcite, accept an electron from free amine, form a radical cation byproduct, and regenerate [Cu(I)/L]^+^ (Scheme 2C). However, this is a minor contribution to activator regeneration under the ZnO photoATRP pathway. Reduction via electron donation by ZnO under photoirradiation was found to be the major contributor, as evidenced above (Table 1, entries 1, 3, and 5) in which free amine donors were not necessary to reach similarly high conversions in 1 h.

Furthermore, the photoATRP pathway exhibited oxygen scavenging directly from the ZnO photocatalyst (Scheme 2B and Figure 3B). The mechanoATRP pathway, like most regenerative ATRP techniques, can undergo slower oxygen removal catalyzed by copper activators, with induction periods on the order of an hour or even more, depending on the amount of ambient reactor headspace. Ordinary oxygen removal in regenerative ATRP proceeds by forming [Cu(I)/L]^+^ through the main reduction process, which is immediately trapped by oxygen to form an oxygen-bearing Cu(II) complex that can decompose in the presence of electron donors, especially amines, back to [Cu(I)/L]^+^.^38,43^ Thus, an explicit, unique oxygen removal pathway for mechanoATRP was shown only for photoATRP. The exceptional oxygen tolerance under UV photoirradiation was further corroborated below by the shorter induction periods and ability to decrease the induction period of mechanoATRP by brief photoirradiation under otherwise identical conditions (Figure 7). It is feasible that ZnO under UV photoirradiation could perform the conventional oxygen removal faster than mechanoATRP, as well as faster reduction of Cu(II) species (i.e., faster cocatalytic reduction of Cu(II) species), but the direct photoinduced removal of oxygen by ZnO is the dominant O_2_ removal mechanism (Figure 3Ba vs 3Bb). However, the two oxygen elimination methods were found to operate in tandem (Figure 3Bd). Thus, ZnO cocatalyst was found to be central to competitive oxygen tolerance.

### 2.6 Kinetics of ZnO-Cocatalyzed PhotoATRP

**Figure 4:**
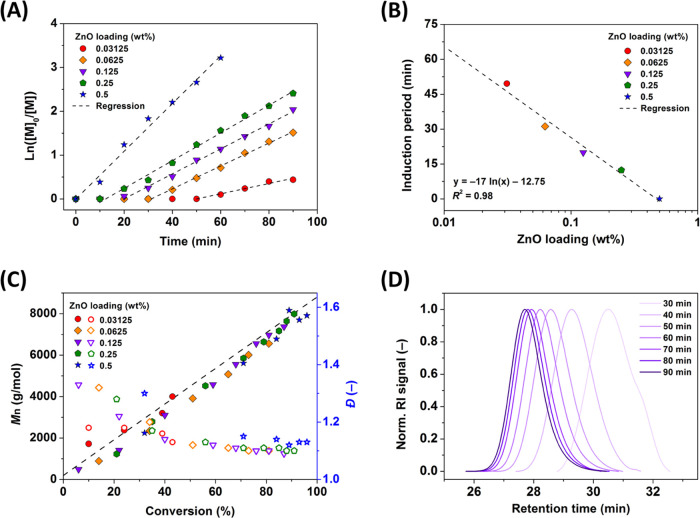
(A) Semilogarithmic kinetic plots of ZnO-cocatalyzed photoATRP of MA with various loadings of ZnO nanocrystals, (B) the dependence of the induction period on ZnO loading, (C) the evolution of molecular weight (solid symbols) and *Đ* (open symbols) with conversion, and (D) the GPC traces at different reaction times (ZnO: 0.125 wt %). Reaction conditions: [MA]_0_/[EBiB]_0_/[CuBr_2_]_0_/[TPMA]_0_ = 100/1/0.04/0.04 in 50% (v/v) DMSO, without prior deoxygenation, closed-cap reactor bearing 20% (v/v) of aerated headspace, under UV irradiation (380 nm, 28.5 mW/cm^2^).

After understanding the complex role of the ZnO nanocrystals in photoATRP with high oxygen tolerance, the effect of the amount of ZnO on the reaction kinetics, with the equimolar Cu(II)/L ratio, was investigated. The initial loading of ZnO was decreased by half after each iteration, i.e., 0.5/*x* wt %, where *x* = 1, 2, 4, 8, or 16. In terms of concentration, the most diluted system (0.03125 wt %) contained only ∼0.32 mg/mL ZnO, making ZnO nanocrystals one of the most effective catalysts in heterogeneous photoATRP. As shown in Figure 4A, polymerizations exhibited first-order kinetics, indicating a constant concentration of radicals being formed,^25^ for all applied ZnO loadings. The polymerization rate was controlled by the amount of the ZnO photocatalyst, giving apparent propagation rate constants, *k*_p_^app^, in a range of 1.16–5.37 × 10^–2^ min^–1^ for the investigated concentrations. A short induction period (extracted from the regression models) was observed and related to the time required for oxygen depletion, following the findings in Figure 3B. The induction period progressively decreased with the higher photocatalyst loading, and it diminished once the ZnO loading approached 0.5 wt %. The induction period scales linearly with −ln[ZnO] (Figure 4B). The molecular weights of poly(methyl acrylate) (PMA) synthesized through ZnO-cocatalyzed photoATRP increased linearly with monomer conversion (Figure 4C), in agreement with theoretical values, *M*_*n*,th_ = *M*_EBiB_ + *M*_MA_ × DP × conv., where *M*_EBiB_ and *M*_MA_ are the molecular weights of the initiator and MA, respectively, and DP is the targeted number-average degree of polymerization, i.e., [MA]_0_/[EBiB]_0_. Polymers with low dispersities (*Đ* ∼1.1) were formed. The GPC traces shown in Figure 4D shifted smoothly to higher molecular weights (lower retention time) without any tailing. GPC traces for other reactions are shown in Figure S3.

### 2.7 Temporal Control of ZnO-Cocatalyzed PhotoATRP

**Figure 5:**
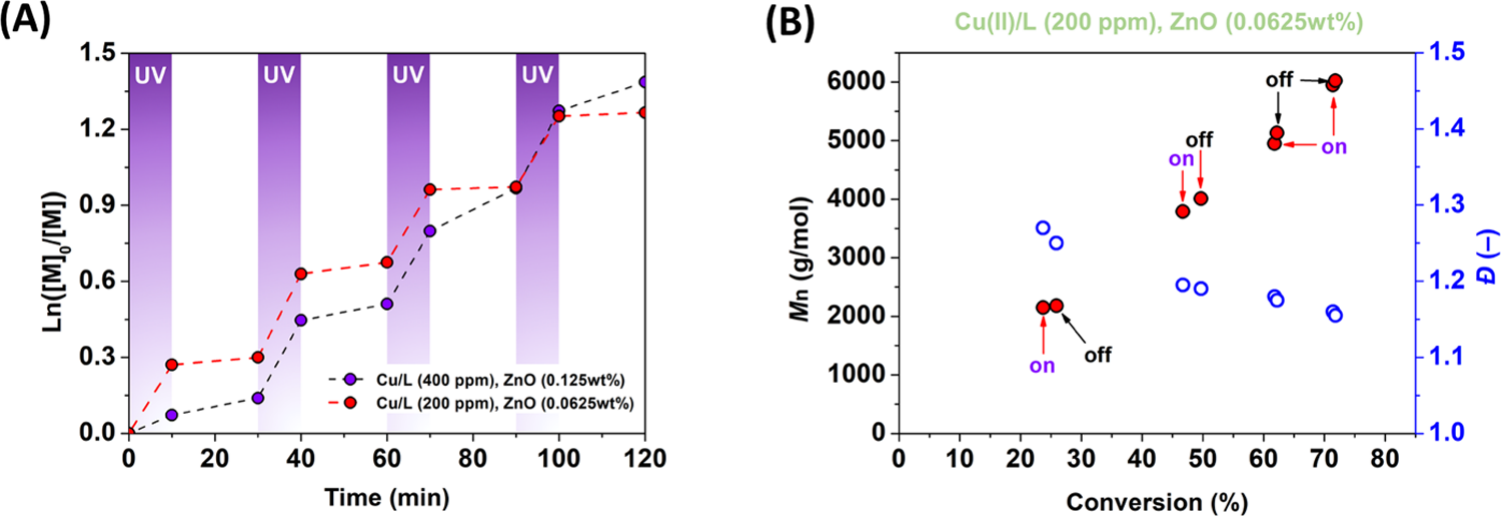
(A) Temporal control and (B) the evolution of molecular weight and *Đ* in ZnO-cocatalyzed photoATRP of MA upon intermittent switching the UV light on/off (380 nm, 28.5 mW/cm^2^). Reaction conditions: [CuBr_2_]_0_/[TPMA]_0_ = 0.04/0.04 and 0.02/0.02 with ZnO loadings of 0.125 and 0.0625 wt %. Time zero represents the end of the induction period in the kinetic experiments.

The advantage of photoATRP is the possibility to control the reaction kinetics externally, by periodic light exposure.^10,13,23,25^ Temporal control of ZnO-cocatalyzed photoATRP showed that the reaction rate can be effectively modulated by on/off switching of the UV light source (Figure 5A). For the initial conditions with [MA]_0_/[EBiB]_0_/[CuBr_2_]_0_/[TPMA]_0_ molar ratios of 100/1/0.04/0.04 photocatalyzed with ZnO (0.125 wt %), the polymerization rate noticeably decreased during the dark phase; however, the chain propagation still continued to some extent (Figure S4). The ongoing process was attributed to a relatively high concentration of the Cu(I)/L activator species engaged in the polymerization.^13^ This phenomenon was successfully suppressed by reducing the amount of [CuBr_2_]_0_/[TPMA]_0_ from 0.04/0.04 to 0.02/0.02 with a subsequent reduction in ZnO loading from 0.125 to 0.0625 wt %, respectively (Figure 5A). The ratio of the apparent propagation rate constants during the UV-off and UV-on cycles (*k*_off_/*k*_on_) decreased from 0.173 to 0.082, demonstrating the enhanced temporal control with a lower concentration of [CuBr_2_]_0_/[TPMA]_0_. A lower concentration of the copper catalyst is usually associated with higher dispersity values due to a slower deactivation rate.^44^ In this sense, a slight increase in dispersity (from *Đ* = 1.11 to 1.16, above 70% conversion) was observed as the Cu(II) levels decreased from 400 to 200 ppm (Figure 5B). Thus, ZnO-cocatalyzed photoATRP can be fine-tuned toward an excellent temporal control under intermittent UV irradiation.

### 2.8 Targeting Various DPs and Expanding Monomer Scope

**Figure 6:**
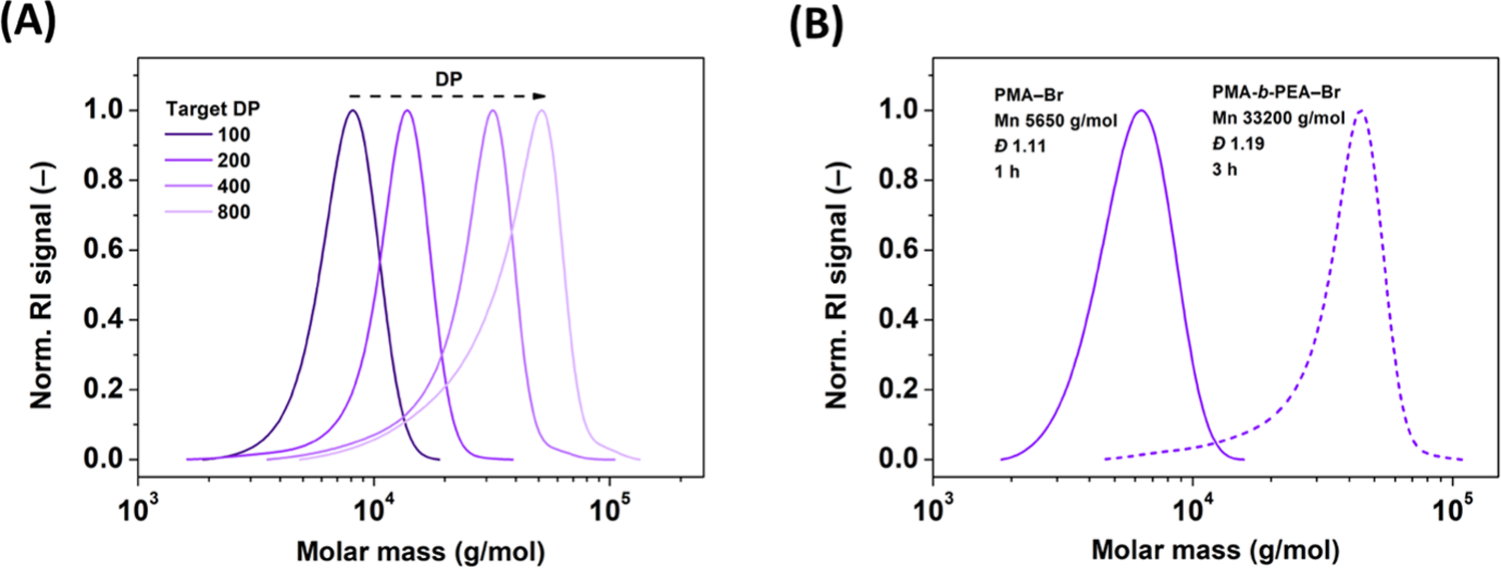
(A) Results of targeting different DP_T_ of PMA. (B) GPC chromatograms of the PMA–Br macroinitiator and the PMA-*b*-PEA–Br copolymer using ZnO-cocatalyzed photoATRP. Block copolymerization conditions: [EA]_0_/[PMA–Br]_0_/[CuBr_2_]_0_/[TPMA]_0_ = 100/0.25/0.04/0.04 with a ZnO loading of 0.125 wt % in 50% (v/v) DMSO, without prior deoxygenation, closed-cap reactor bearing 20% (v/v) of aerated headspace, under UV irradiation (380 nm, 28.5 mW/cm^2^).

ZnO-cocatalyzed photoATRP to target different degrees of polymerization (DP) with hydrophobic (meth)acrylate monomers was investigated. The results are summarized in Table 2 and Figure S5. The loadings of the monomer, the [Br–Cu(II)/TPMA]^+^ complex, and DMSO were kept constant while adjusting the targeted DP through the concentration of EBiB. The high DP values (up to DP_T_ 800) were accessible for PMA with relatively low dispersity values, ranging from 1.10 to 1.28 (Table 2, entries 1–4 and Figure 6A). When targeting higher DP_T_, the apparent molar mass, *M*_*n*,GPC_, was notably lower than the theoretical one, *M*_*n*,th_. This indicates the generation of new chains during UV irradiation. PhotoATRPs of ethyl acrylate (EA) and 2-hydroxyethyl acrylate (HEA, DP = 100 and 400) were also highly controlled, with a reaction rate comparable to that of MA (Table 2, entries 5–8). A more pronounced difference between *M*_*n*,th_ and *M*_*n*,GPC_, in the case of HEA, could be attributed to HMW shouldering due to the presence of cross-linker impurities and the difference between the hydrodynamic volumes of poly(2-hydroxyethyl acrylate) (PHEA) and PMMA calibration standards. The polymerization of methyl methacrylate (MMA) was less controlled, yielding polymers with higher dispersities (Table 2, entries 9–10).

**Table 2:** Results of ZnO-Cocatalyzed PhotoATRPs with Different Monomers Targeting Various DPs

entry^a^	monomer	DP_T_	time (h)	conv.^b^ (%)	*M*_*n*,th_^c^	*M*_*n*,GPC_^d^	*Đ*^d^ (−)
1	MA	100	1.5	86	7600	7240	1.10
2		200	1.5	77	13,450	11,740	1.13
3		400	1.5	81	28,090	23,960	1.20
4		800	1.5	72	49,780	33,240	1.28
5	EA	100	1.5	95	9710	10,870	1.11
6		400	1.5	84	33,830	32,620	1.15
7	HEA	100	1.5	86	10,180	15,300^e^	1.14
8		400	1.5	55	25,740	41,560^e^	1.16
9	MMA	100	1.5	48	5010	8240	1.27
10		400	1.5	49	19,820	18,210	1.41

a Reaction conditions: [monomer]_0_/[EBiB]_0_/[CuBr_2_]_0_/[TPMA]_0_ = 100/*y*^–1^/0.04/0.04, where *y* = 1, 2, 4, and 8 in 50% (v/v) DMSO without prior deoxygenation, closed-cap reactor bearing 20% (v/v) of aerated headspace, loading of the ZnO nanocrystals (0.125 wt %), UV light source (380 nm, 28.5 mW/cm^2^), ambient temperature.

b Determined from ^1^H NMR spectra (CDCl_3_ as the solvent).

c Calculated following the equation *M*_*n*,th_ = *M*_EBiB_ + [MA]_0_/[EBiB]_0_ × conversion × *M*_MA_.

d Determined by GPC analysis (THF as the eluent) calibrated to linear PMMA standards.

e Determined by GPC analysis (DMF as the eluent) calibrated with linear PMMA standards.

### 2.9 Chain-End Functionality

To confirm the chain-end functionality of the polymers prepared by this method, the PMA–Br macroinitiator was synthesized under the optimized conditions (Section 1.10 in the SI). After purification, the PMA–Br homopolymer (conv. 55%, *M_n_* = 5,650, *Đ* = 1.11, DP_T_ = 100) was used for polymer extension with EA, resulting in a diblock copolymer, PMA-*b*-PEA–Br (conv. 71%, *M_n_* = 33,200, *Đ* = 1.19, DP_T_ 400). As displayed in Figure 6B, the GPC peak for the copolymer clearly shifted toward higher molecular weights, without any “shoulder” from the unreacted macroinitiator. The results imply a high chain-end functionality and a low fraction of terminated chains. It is noteworthy that a similar copolymer (PMA-*b*-PEA–Br, 35,500) was recently prepared by sonochemistry-assisted ATRP in degassed reactors with 0.45 wt % loading of the manganese carbonyl, using 6-fold excess of ligand.^45^ By using ZnO-cocatalyzed photoATRP, such a copolymer was obtained under the equimolar loading of CuBr_2_ to the ligand in nondeoxygenated reactors in just 3 h with 0.125 wt % loading of the ZnO nanocrystals.

### 2.10 Synergistic Aspects

As shown, ZnO-cocatalyzed photoATRP performed under an equimolar CuBr_2_/L concentration provided superior kinetics to analogous mechanoATRP of acrylates (Table 1). Mechanically controlled reactions (FRP, ATRP, and RAFT), however, reported the fabrication of HMW polymers, polymeric gels,^46^ resins,^32^ or self-strengthening (bio)composites,^47^ due to the penetration depth of the ultrasound. For these reasons, we sought to combine both techniques, creating a robust methodology that is endowed with synergistic utilization of fast deoxygenation and a deep penetration limit.

**Figure 7:**
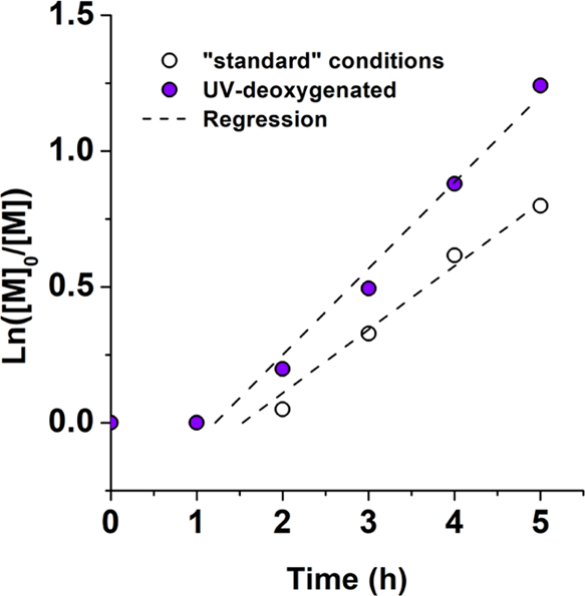
Semilogarithmic kinetic plots of ZnO-cocatalyzed (0.5 wt %) mechanoATRP of MA. The “standard” reactions (open symbols), and the reactions with prior UV deoxygenation (solid symbols). Reaction conditions: [MA]_0_/[EBiB]_0_/[CuBr_2_]_0_/[TPMA]_0_ = 100/1/0.04/0.16 in 50% (v/v) DMSO, ultrasound source (40 kHz, 110 W).

Since ZnO-cocatalyzed mechanoATRP showed a long induction period (∼90 min, Table 1), we utilized the deoxygenation ability of the UV treatment from ZnO-cocatalyzed photoATRP (Figure 3B) to successfully diminish the induction period before conducting the mechanoATRP process. Therefore, the reaction mixture was shortly irradiated (3 min), followed by “standard” ZnO-cocatalyzed mechanoATRP. As shown in Figure 7, the reaction deoxygenated under UV light exhibited a decreased induction period and a faster reaction (*k*_p_^app^ of 0.39 × 10^–2^ vs 0.53 × 10^–2^ min^–1^). This, however, indicated that the presence of oxygen was not a single factor responsible for the induction period. Since both mechanoATRP reactions were inactive for 1–1.5 h of sonication, it was assumed that the catalytic activity of ZnO was likely hindered due to the fouling of the attached surfactant.^48^ To remove the capping agent, ZnO nanocrystals were exposed to sonication (2 h) before being injected into the ATRP cocktail. Such a treatment resulted in a further diminished induction period and a faster reaction of mechanoATRP (Figure S6); further improvements were achieved by using UV-assisted deoxygenation (*k*_p_^app^ of 0.69 × 10^–2^ vs 0.77 × 10^–2^ min^–1^). A control experiment was performed and the presence of the detached surfactant in the supernatant was detected by ^1^H NMR spectroscopy (Figure S7). The spectrum corresponded to that of neat oleic acid (OA), but the intensity of the double-bond signals, at a position of 5.30 ppm was diminished. This suggested that OA had been removed from the ZnO surface but also undergone chemical transformation during ultrasonication.^49^ It can be concluded that both techniques can be synergistically combined to enable a more robust ATRP methodology devoid of dedicated degassing such as FPT or sparging. Also, the effects of surfactants have to be taken into consideration when designing new heterogeneous mechano/photoATRP systems.

## 3 Conclusions

In summary, wurtzite ZnO nanocrystals were employed as universal agents for mechano- and photoATRP. Polymerization kinetics was superior under UV irradiation, even leading to fast photoATRP that proceeded without excess ligand, i.e., under an equimolar Cu(II)/L concentration and very low ZnO loadings (>0.32 mg/mL). Activator regeneration via electron donation by ZnO was the major contributor under photoirradiation compared to the classical photoATRP process. ATRP was carried out in partly aerated reactors without prior deoxygenation due to the strong oxygen scavenging capability of photoirradiated ZnO. Polymers with predictable molecular weights and low dispersity were prepared from various (meth)acrylates. Polymer growth was easily modulated by switching the UV light on/off, and a high chain-end functionality provided block copolymers. The polymerization kinetics of mechanoATRP increased in rate when ZnO aggregates were more thoroughly homogenized and the surfactant was cleaved off by pretreating the ZnO cocatalyst with ultrasonication. ZnO-cocatalyzed photoATRP is a promising technique due to its fast reaction rate, high oxygen tolerance, minimal use of ligands, low ZnO photocatalyst loadings, and facile separation of heterogeneous ZnO.

## Supporting Information Available

List of materials and experimental details; DRUV–vis spectrum and Tauc’s plot for ZnO nanocrystals; detailed data for the control ATRP experiments; NMR spectra for DMSO_2_ monitoring; additional GPC traces for photoATRP experiments; additional temporal control data; GPC traces of different polymers prepared by ZnO-cocatalyzed photoATRP; kinetic plots after ZnO preconditioning; detection of detached oleic acid by NMR; and detailed discussion about the mechanism for ZnO-cocatalyzed photoATRP (PDF)
