# Supplementary material for: Comparison of Mechano- and PhotoATRP with ZnO Nanocrystals
Source: Macromolecules. 2023 Jun 26;56(13):5101–10. doi: 10.1021/acs.macromol.3c00250 (PMC10339823; doi:10.1021/acs.macromol.3c00250)
Supplement: Supplementary file 1 — ma3c00250_si_001.pdf [file ma3c00250_si_001.pdf]

Supplementary Information

Comparison of mechano- and photoATRP with ZnO nanocrystals

Martin Cvek,[a,b] Arman Moini Jazani,[a] Julian Sobieski,[a] Thaiskang Jamatia,[b]
Krzysztof Matyjaszewski[a,]*

a) Department of Chemistry, Carnegie Mellon University, 4400 Fifth Avenue, Pittsburgh, Pennsylvania 152 13, United States of America

b) Centre of Polymer Systems, Tomas Bata University in Zlin, Trida T. Bati 5678, 760 01 Zlin, Czech Republic

*Author to whom correspondence should be addressed: km3b@andrew.cmu.edu

Table of Contents

1. Experimental Part 3

1.1. Materials 3

1.2. Synthesis of stabilized ZnO nanocrystals 3

1.3. General procedure for the ZnO co-catalyzed ATRP 4

1.4. Oxygen concentration measurements 4

1.5. Detection of dimethyl sulfone 4

1.6. Monitoring of the ZnO co-catalyzed photoreduction 5

1.7. Kinetics of the ZnO co-catalyzed photoATRP 5

1.8. Temporal control of the ZnO co-catalyzed photoATRP 5

1.9. Tuning degree of polymerization 6

1.10. Synthesis of diblock copolymer 6

1.11. Detachment of oleic acid after sonication 6

1.12. Characterizations 7

2. Results and Discussion 8

Figure S1. (**A**) DRUV-Vis absorbance spectrum of the ZnO nanocrystals, and (**B**) the determination of their band gap energy from the Tauc's plot. 8

Supplementary Information

Table S1. Reference photoATRPs (without ZnO); the effect of the Cu(II)/L on the occurrence of side reactions. 8

Table S2. Control experiments in the ZnO co-catalyzed photoATRP of methyl acrylate. 8

Table S3. Effect of the $CuBr_2$ concentration on the ZnO co-catalyzed photoATRP of methyl acrylate. 9

Figure S2. Monitoring of $DMSO_2$ evolution by NMR spectroscopy (acetone-d6). Reaction conditions: dispersion of ZnO nanocrystals (0.25 wt%) in DMSO, under UV irradiation (380 nm, 28.5 mW/cm^2). (**A**) Fresh solution, (**B**) closed reactor irradiated for 30 min, (**C**) open reactor irradiated for 60 min (total time 90 min), and (**D**) open reactor irradiated for 120 min (total time 150 min). The initial presence of $DMSO_2$ in the fresh solution is considered as an impurity. 9

Figure S3. GPC traces at different reaction times for the ZnO co-catalyzed photoATRPs with different (**A**, **B**, **C** and **D**) loading of the ZnO nanocrystals (0.5/*x* wt%, where *x* = 1, 2, 8 and 16). Reaction conditions: $[MA]_0/[EBiB]_0/[CuBr_2]_0/[TPMA]_0$ = 100/1/0.04/0.04 in 50% (v/v) DMSO, without deoxygenation, closed-cap reactor bearing 20% (v/v) of aerated headspace, under UV irradiation (380 nm, 28.5 mW/cm^2). 10

Figure S4. Temporal control in the ZnO co-catalyzed photoATRP of the MA upon intermittent switching on/off the UV light (380 nm, 28.5 mW/cm^2), expressed as the time evolution of molecular weight and *Đ* of the PMA. Reaction conditions: $[CuBr_2]_0/[TPMA]_0$ = 0.04/0.04 with the ZnO loading of 0.125 wt%. 10

Figure S5. GPC traces of different polymers prepared by the ZnO co-catalyzed photoATRP with 0.125 wt% loading of the ZnO nanocrystals. Reaction conditions: $[monomer]_0/[EBiB]_0/[CuBr_2]_0/[TPMA]_0$ = 100/0.25/0.04/0.04 in 50% (v/v) DMSO, without deoxygenation, closed-cap reactor bearing 20% (v/v) of aerated headspace, under UV irradiation (380 nm, 28.5 mW/cm^2). 11

Figure S6. Semilogarithmic kinetic plots of ZnO co-catalyzed (0.5 wt%) mechanoATRP of MA after ZnO pre-conditioning. The "standard" reactions (*open symbols*), the reactions with prior UV-deoxygenation (*solid symbols*). Reaction conditions: $[MA]_0/[EBiB]_0/[CuBr_2]_0/[TPMA]_0$ = 100/1/0.04/0.16 in 50% (v/v) DMSO; ultrasound source (40 kHz, 110 W). 11

Figure S7. ^{1}H NMR spectra of (**A**) starting DMSO, and (**B**) DMSO supernatant after 4 hours of sonicating dispersion of ZnO (50 mg/mL). The region of expected double bond assignment is denoted. 12

2.1. Discussion of the electron donor in the absence of excess TPMA for ZnO co-catalyzed photoATRP 12

3. References 14

Supplementary Information

1. Experimental Part

1.1. Materials

All chemicals and reagents were sourced from commercially available suppliers and used as received, unless stated otherwise. Zinc acetate dihydrate (ZAD, p.a.), diethylene glycol (DEG, ≥99.0%, Reagent Grade), oleic acid (OA, ≥99.0%), methanol (≥99.6%), methyl acrylate (MA, 99%, ≤100 ppm of monomethyl ether hydroquinone, MeHQ), ethyl acrylate (EA, 99%, 15–20 ppm of MeHQ), 2-hydroxyethyl acrylate (HEA, 96%, 200–650 ppm of MeHQ), methyl methacrylate (MMA, 99%, ≤30 ppm of MeHQ) and ethyl-α-bromoisobutyrate (EBiB, 98%), copper(II) bromide ($CuBr_2$, 99%) were obtained from Sigma Aldrich (USA). Monomers were passed through the column of aluminum oxide (activated, basic, Brockmann I) to remove the MeHQ inhibitors prior their use. Tris(2-pyridylmethyl)amine (TPMA) was purchased from AmBeed (USA). Dimethyl sulfoxide (DMSO, ≥99.7%) and tetrahydrofuran (THF, 99.9%) were supplied by Fisher Scientific (USA), d-chloroform ($CDCl_3$, 99.8%), acetone-d6 (99.9%) and dimethyl sulfoxide-d6 (DMSO-d6, 99.9%) were products of Cambridge Isotope Laboratories (USA).

1.2. Synthesis of stabilized ZnO nanocrystals

The ZnO nanocrystals were synthesized using a facile microwave (MW)-assisted polyol technique, after modifying protocols reported by Jamatia et al.[1] and Hammarberg et al.[2] In brief, a Teflon-lined reactor was charged with the ZAD powder (32 mmol, 7.02 g) representing a precursor, which was dispersed in 50 mL of DEG serving as a medium. Afterwards, the capping agent, i.e. OA (2.3 mmol, 0.660 g), was introduced into the mixture, followed by the additional stirring for 10 minutes. The Teflon vessel was sealed and placed into the MW reactor (Magnum II, Ertec, Poland), which was set to 100% intensity with the reaction time of 15 minutes. During the defined synthesis program, temperature and pressure steeply increased and plateaued at 245–250°C and 45–50 bar, respectively. Then, the reactor vessel was cooled down to 45°C using water circuit. The ZnO nanocrystals were separated using centrifugation (EBA 21 Hettich, Germany) (5 min, 6000 rpm), rinsed several times with methanol, and dried at 65°C under 150 mbar overnight.

1.3. General procedure for the ZnO co-catalyzed ATRP

In a typical procedure,[3] the ATRP reactants, MA (1.92 mL, 21.2 mmol, 100 eq.), EBiB (31.1 µL, 0.21 mmol, 1 eq.), TPMA (0.04–0.16 eq.; specified further in text, 2.5–9.8 mg, 8.5–33.9 µmol), $CuBr_2$ (1.9 mg, 8.5 µmol, 0.04 eq.) and DMSO (50 vol.%) were sequentially dosed into the reaction flask which was fitted with a magnetic stir bar. The ZnO loading constituted 0.5/*x* wt% (*x* = 1, 2, 4, 8, or 16), relative to the monomer (MA) and solvent (DMSO) weight. A good dispersion of the components was aided by a vortex mixer (Fisher Scientific, USA). The reference reactions were performed in the absence of the ZnO nanocrystals. The vial for mechanoATRP was exposed to the shock-waves in an ultrasonic bath CPX2800H (40 kHz, 110W, Bransonic, Mexico) with temperature below 30°C (excitation parameters commonly used for conducting piezoelectrically mediated reactions),[4] its analogue intended for photoATRP was placed into air-conditioned PhotoRedOx box (HepatoChem, USA) fitted with the ultraviolet (UV) light source (380 nm, 28.5 mW/cm^2, HepatoChem, USA). Irradiance at the position of the vial was measured using a PM100D optical power meter (ThorLabs, Germany). All reactions were performed in 4.8 mL closed-cap vials (having aerated headspace of approx. 20% (v/v)), without applying deoxygenation techniques, thus, the volume of the reaction mixture represented 3.84 mL.

1.4. Oxygen concentration measurements

The real-time oxygen concentration inside the reactor was measured using a FireStingGO2 pocket oxygen meter (PyroScience, Germany) equipped with the solvent-resistant fiber-optic sensor. The needle-like probe was inserted into the reactor through the septum, and the concentration of dissolved oxygen was recorded as a function of time, upon UV irradiation (380 nm, 28.5 mW/cm^2).

1.5. Detection of dimethyl sulfone

A dispersion of ZnO nanocrystals (0.25 wt%) in DMSO was placed into a reactor (bearing aerated headspace of approx. 20% (v/v)) and irradiated by UV light (380 nm, 28.5 mW/cm^2). The experiment started with the closed reactor. The reactor was opened after 30 min of irradiation, and the reaction followed by additional 120 min. The aliquots of the reaction mixture (100 µL) were mixed with acetone-d (650 µL); toluene (30 µL) was added as a reference. A formation of dimethyl sulfone ($DMSO_2$) was monitored by ^{1}H NMR technique.

1.6. Monitoring of the ZnO co-catalyzed photoreduction

The ZnO-mediated photoreduction of the $CuBr_2$/TPMA was monitored using the UV-Vis-NIR spectrophotometer (Cary 5000, Agilent Technologies, USA). The reaction solution was transferred into a quartz glass cuvette charged with a small magnetic stirrer, and sealed using the fitted rubber septum. The cuvette with the solution was irradiated by UV light (380 nm, 28.5 mW/cm^2) and the absorption spectra were collected in predetermined intervals. The photoreduction of the $CuBr_2$/TPMA was evaluated from the absorbance decrease at 945 nm, similarly, as reported by Wang et al.[5] Due to high turbidity of caused by the ZnO nanocrystals, the experiment was made using TPMA (0.04 eq.), $CuBr_2$ (0.04 eq.) in DMSO with the lowest effective ZnO loading of 0.5/*x* wt%, where *x* = 16 (i.e. 0.03125 wt%).

1.7. Kinetics of the ZnO co-catalyzed photoATRP

The ATRP mixtures with different loadings of the ZnO nanocrystals (0.5/*x* wt%, where *x* = 1, 2, 4, 8, or 16) were prepared following the general procedure (Section 1.3.). For this type of reactions, the protocol with the equimolar Cu(II)/L ratio was selected. Thus, the final concentrations were fixed to: MA (100 eq.), EBiB (0.01 eq.), TPMA (0.04 eq.), $CuBr_2$ (0.04 eq.) and DMSO (50 vol.%). To ensure the accuracy, the reagents were dosed from the fresh stock solutions. The samples (100 µL) were withdrawn through the rubber sealing (without opening the reactor) with a period of 10 min, and analyzed using ^{1}H NMR and GPC techniques.

1.8. Temporal control of the ZnO co-catalyzed photoATRP

The ATRP mixture was prepared following the aforementioned protocol (Section 1.3. and Section 1.7.), while using the ZnO loading of 0.5/*x* wt%, where *x* = 4 (i.e. 0.125 wt%); calculated relative to the monomer (MA) and solvent (DMSO) weight. The reaction kinetics was modulated by intermittent switching on/off the UV light source (380 nm, 28.5 mW/cm^2). Each irradiation phase lasted 10 min., followed by the dark phase of 20 min. The samples (100 µL) were taken at the corresponding intervals, and analyzed using ^{1}H NMR and GPC techniques. Note that the first irradiation phase was adjusted to the induction time that was determined based on the linear regression model applied in the kinetics experiment (Section 1.7.).

1.9. Tuning degree of polymerization

The following reaction was selected as a model example for targeting the various degrees of polymerization (DP_T). The reactants comprised the following: MA (100 eq.), EBiB (0.01 eq.), TPMA (0.04 eq.), $CuBr_2$ (0.04 eq.) and DMSO (50 vol.%), with the ZnO loading of 0.5/*x* wt%, where $x = 4$ (i.e. 0.125 wt%); calculated relative to the monomer (MA) and solvent (DMSO) weight. The concentrations of all reactants were fixed, while the concentration of EBiB was varied (y^{-1} eq., where $y = 1$, 2, 4, 8), which resulted to DP_T of 100, 200, 400 and 800, respectively. The reactors (specified in Section 1.3.) were irradiated by UV light (380 nm, 28.5 mW/cm^2), the polymerizations were stopped after 90 min, and the products were analyzed using ^{1}H NMR and GPC techniques.

1.10. Synthesis of diblock copolymer

The PMA macroinitiator (PMA-Br) was prepared following the general procedure for the ZnO co-catalyzed photoATRP (Section 1.3.) with the MA (100 eq.), EBiB (0.01 eq.), TPMA (0.04 eq.), $CuBr_2$ (0.04 eq.) and DMSO (50 vol.%) and the ZnO loading of 0.5/*x* wt%, where $x = 4$ (i.e. 0.125 wt%). The polymerization was stopped at a conversion of 55% with *M*n of 5690 g/mol and *Đ* of 1.11. The catalytic system and the ZnO nanocrystals were removed by passing the ATRP mixture through the column of alumina. Unreacted monomer and solvent were evaporated using an evaporation system (Biotage V-10 Touch, Sweden) and the intense air flow. To execute the chain extension, PMA-Br macroinitiator (0.25 eq.) was applied in a fresh ATRP solution consisting of EA (100 eq.), TPMA (0.04 eq.), $CuBr_2$ (0.04 eq.) and DMSO (50 vol.%) with the ZnO loading of 0.5/*x* wt%, where $x = 4$ (i.e. 0.125 wt%), thereby resulting in DP_T of 400. The reactor was irradiated by UV light (380 nm, 28.5 mW/cm^2) for additional 120 minutes, and resulting PMA-*b*-PEA-Br copolymer was analyzed by ^{1}H NMR and GPC techniques.

1.11. Detachment of oleic acid after sonication

A dispersion of OA-capped ZnO nanocrystals in DMSO-d6 (50 mg/mL) was sonicated (40 kHz, 110W) at a temperature below 30°C for 4 hours. Subsequently, ZnO was allowed to settle down, and the supernatant was analyzed by ^{1}H NMR spectroscopy.

1.12. Characterizations

The morphology of ZnO nanocrystals was investigated using the TEM performed on a JEM-2100Plus (JEOL, Japan) equipped with the LaB6 emission source, operating at the accelerating voltage of 200 kV. The mean dimensions of the nanocrystals were acquired from the analysis of multiple TEM images, using ImageJ software (version 1.52a, National Institutes of Health, USA).

The crystallinity was assessed from the XRD patterns collected on a Miniflex 600 (Rigaku, Japan) using Co-Kα radiation source (λ = 1.789 Å) operating within the 2θ range from 20° to 100°, at a scan speed of 3°/min.

UV-Vis diffuse reflectance (DRUV-Vis) spectra were recorded on a Lambda 1050 (Perkin-Elmer, USA) spectrophotometer in the range from 300 to 800 nm to study the relevant optical properties, such as absorbance and the band gap energy, of the ZnO nanocrystals. The latter was determined from DRUV-Vis spectra using the Kubelka-Munk function, *F*(*R*):

$$F(R) = \frac{(1-R)^2}{2R}$$

where *R* is the absolute reflectance of the ZnO nanocrystals. Tauc's plot relating the $[F(R)\times h\nu]^2$ against photon energy, $h\nu$, where h is the Planck's constant and ν is the frequency of light, was used to determine the value of the optical band gap energy. The extrapolation of the "on-set" spectral straight line to $h\nu$ = 0 gave the value of band gap energy.

The monomer conversion was determined from ^{1}H NMR spectra (32 scans), which were recorded on 500 MHz Avance III (Bruker, USA) spectrometer using deuterated solvents (specified above) at the ambient temperature.

The molecular weights and the dispersity index (*Đ*) were calculated from GPC traces collected on 1230 Infinity II (Agilent Technologies, USA) equipped with the Waters 515 HPLC pump, PSS columns (Styrogel 105, 103, 102Å) and Water 2414 refractive index (RI) detector. THF as an eluent at the flow rate of 1 mL/min and a temperature of 35°C. In the case of poly(hydroxyethyl acrylate) (PHEA), DMF was used as an eluent at the flow rate of 1 mL/min and a temperature of 50°C. The calibration of GPCs was performed using PMMA standards and the data was processed using WinGPC 7.0 software.

2. Results and Discussion

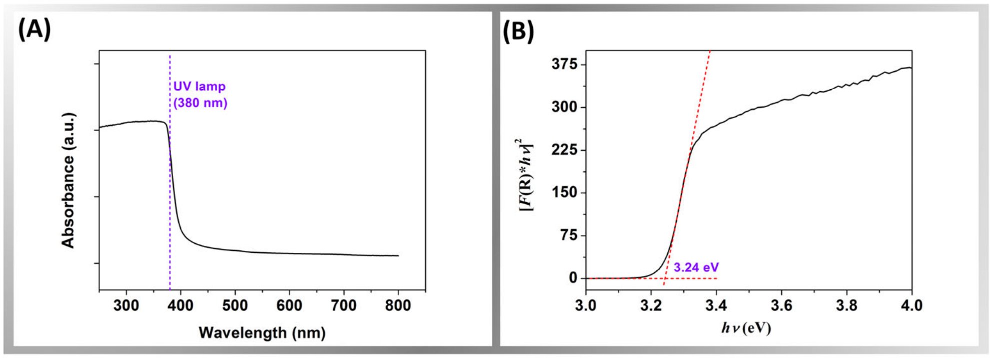

Figure S1. (**A**) DRUV-Vis absorbance spectrum of the ZnO nanocrystals, and (**B**) the determination of their band gap energy from the Tauc's plot.

Table S1. Reference photoATRPs (without ZnO); the effect of the Cu(II)/L on the occurrence of side reactions.

Entry[a]	ZnO (wt%)	Stimulus	$CuBr_2$ (eq.)	TPMA (eq.)	Time (h)	Conv.[b] (%)	$M_{n,th}$[c]	$M_{n,GPC}$[d]	$Đ$[d] (–)
1	0.0	UV	0.04	0.16	1	0	–	–	–
					3	13	–	1050	1.25
					5	32	–	2340	1.14
2	0.0	UV	0.04	0.08	1	0	–	–	–
					3	6	–	< 500	N/D
					5	19	–	1690	1.12
3	0.0	UV	0.04	0.04	1	0	–	–	–
					3	0	–	–	–
					5	< 3	–	< 500	N/D

[a]Reaction conditions: $[MA]_0/[EBiB]_0/[CuBr_2]_0/[TPMA]_0$ = 100/1/0.04/0.04–0.16 in 50% (v/v) DMSO without prior deoxygenation, closed-cap reactor bearing 20% (v/v) of aerated headspace; UV light source (380 nm, 28.5 mW/cm^2), ambient temperature. [b]Determined from ^1H NMR spectra ($CDCl_3$ as solvent). [c]Not defined. [d]Determined by GPC analysis (THF as eluent) calibrated to a linear PMMA standard.

Table S2. Control experiments in the ZnO co-catalyzed photoATRP of methyl acrylate.

Entry[a]	ZnO (wt%)	Stimulus	EBiB (eq.)	$CuBr_2$ (eq.)	TPMA (eq.)	Time (h)	Conv. (%)	$M_{n,GPC}$[c]	$Đ$[c] (–)
1	0.25	UV	1	–	–	1	Gel-like	N/D	N/D
2	0.25	UV	–	0.04	0.04	1	36[b]	12570	1.62
3	0.25	UV	1	0.04	0.04	1	81[b]	7170	1.11

[a]Reaction conditions: with MA (100 eq.) in 50% (v/v) DMSO without prior deoxygenation, closed-cap reactor bearing 20% (v/v) of aerated headspace; UV light source (380 nm, 28.5 mW/cm^2), ambient temperature. [b]Determined from ^1H NMR spectra ($CDCl_3$ as solvent). [c]Determined by GPC analysis (THF as eluent) calibrated to a linear PMMA standard.

Table S3. Effect of the $CuBr_2$ concentration on the ZnO co-catalyzed photoATRP of methyl acrylate.

Entry[a]	ZnO (wt%)	Stimulus	$CuBr_2$ (eq.)	TPMA (eq.)	Time (h)	Conv.[b] (%)	$M_{n,th}$[c]	$M_{n,GPC}$[d]	$Đ$[d] (–)
1	0.25	UV	0.04	0.04	1	85	7510	8030	1.11
					2	97	8550	9160	1.10
2	0.25	UV	0.02	0.02	0.5	89	7860	7050	1.20
					1	98	8630	7750	1.20
3	0.125	UV	0.02	0.02	0.5	78	6910	5970	1.18
					1	94	8290	7600	1.16
4	0.125	UV	0.01	0.01	0.5	95	8370	8370	1.69
					1	99	8720	8720	1.71
5	0.0625	UV	0.01	0.01	0.5	76	6740	6090	1.28
					1	96	8460	7510	1.26

[a]Reaction conditions: $[MA]_0/[EBiB]_0/[CuBr_2]_0/[TPMA]_0$ = 100/1/0.01–0.04/0.01–0.04 in 50% (v/v) DMSO without prior deoxygenation, closed-cap reactor bearing 20% (v/v) of aerated headspace; UV light source (380 nm, 28.5 mW/cm^2), ambient temperature; vial volume of 2 mL. [b]Determined from ^{1}H NMR spectra ($CDCl_3$ as solvent). [c]Calculated following the equation ($M_{n,th} = M_{EBiB} + [MA]_0/[EBiB]_0 \times \text{conversion} \times M_{MA}$). [d]Determined by GPC analysis (THF as eluent) calibrated to a linear PMMA standard.

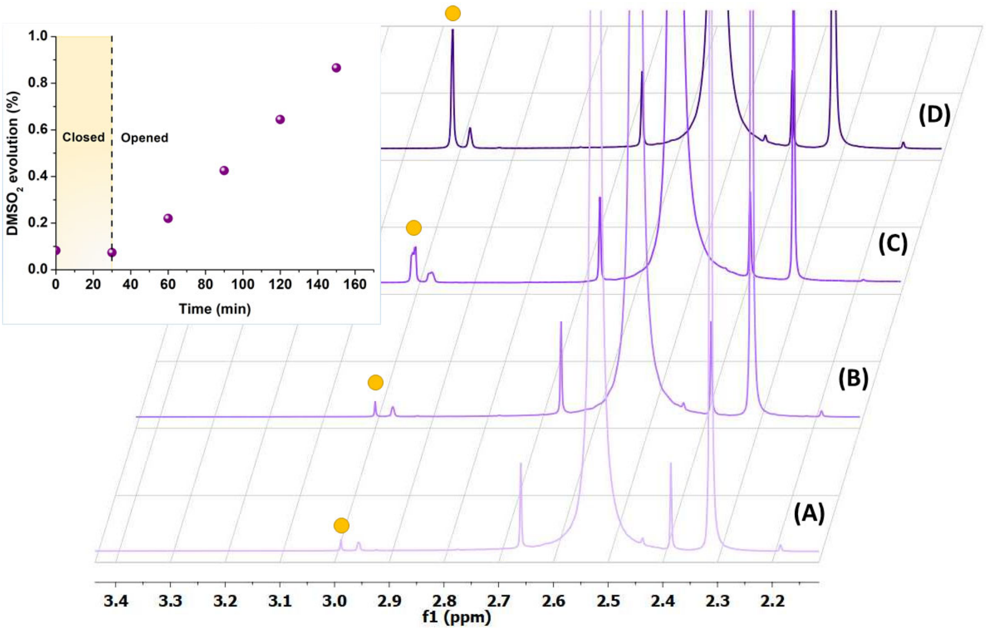

Figure S2. Monitoring of $DMSO_2$ evolution by NMR spectroscopy (acetone-d6). Reaction conditions: dispersion of ZnO nanocrystals (0.25 wt%) in DMSO, under UV irradiation (380 nm, 28.5 mW/cm^2). (**A**) Fresh solution, (**B**) closed reactor irradiated for 30 min, (**C**) open reactor irradiated for 60 min (total time 90 min), and (**D**) open reactor irradiated for 120 min (total time 150 min). The initial presence of $DMSO_2$ in the fresh solution is considered as an impurity.

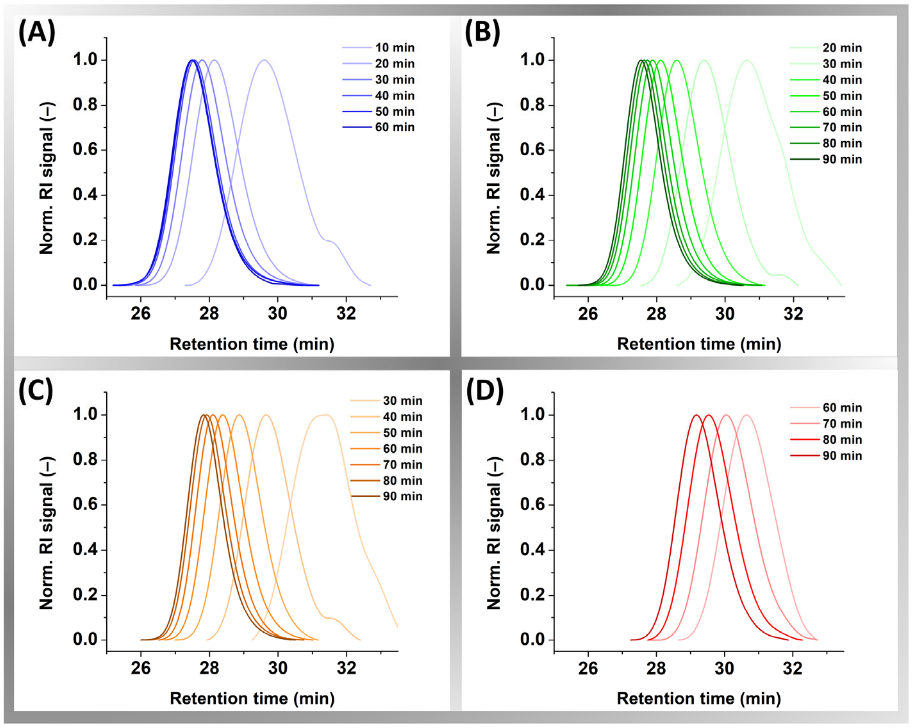

Figure S3. GPC traces at different reaction times for the ZnO co-catalyzed photoATRPs with different (**A**, **B**, **C** and **D**) loading of the ZnO nanocrystals (0.5/*x* wt%, where *x* = 1, 2, 8 and 16). Reaction conditions: $[MA]_0/[EBiB]_0/[CuBr_2]_0/[TPMA]_0$ = 100/1/0.04/0.04 in 50% (v/v) DMSO, without deoxygenation, closed-cap reactor bearing 20% (v/v) of aerated headspace, under UV irradiation (380 nm, 28.5 mW/cm^2).

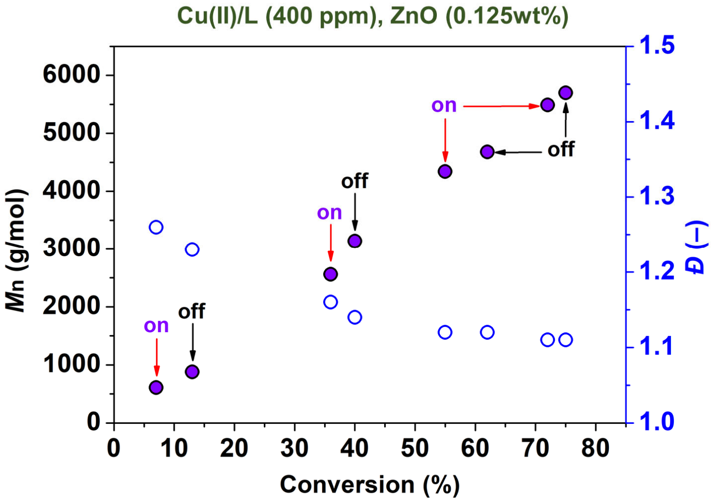

Figure S4. Temporal control in the ZnO co-catalyzed photoATRP of the MA upon intermittent switching on/off the UV light (380 nm, 28.5 mW/cm^2), expressed as the time evolution of molecular weight and *Đ* of the PMA. Reaction conditions: $[CuBr_2]_0/[TPMA]_0$ = 0.04/0.04 with the ZnO loading of 0.125 wt%.

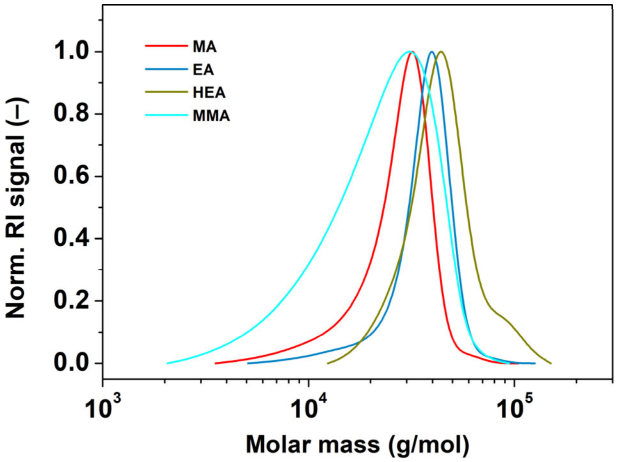

Figure S5. GPC traces of different polymers prepared by the ZnO co-catalyzed photoATRP with 0.125 wt% loading of the ZnO nanocrystals. Reaction conditions: $[monomer]_0/[EBiB]_0/[CuBr_2]_0/[TPMA]_0$ = 100/0.25/0.04/0.04 in 50% (v/v) DMSO, without deoxygenation, closed-cap reactor bearing 20% (v/v) of aerated headspace, under UV irradiation (380 nm, 28.5 mW/cm^2).

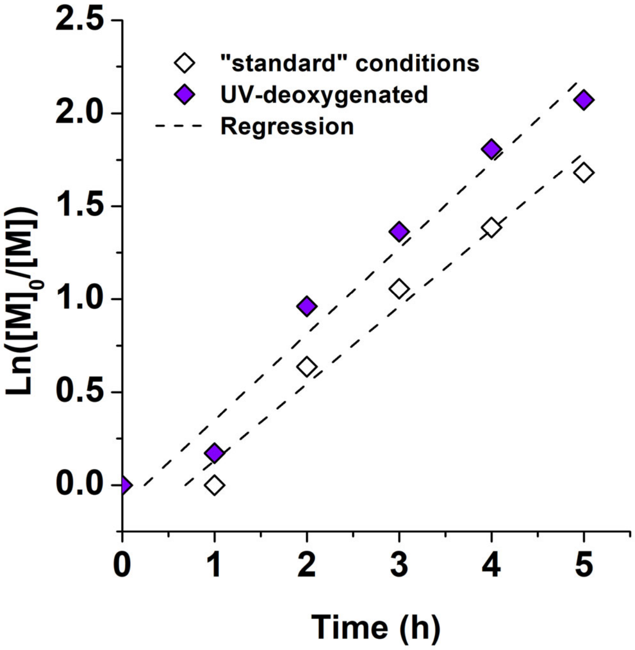

Figure S6. Semilogarithmic kinetic plots of ZnO co-catalyzed (0.5 wt%) mechanoATRP of MA after ZnO pre-conditioning. The "standard" reactions (*open symbols*), the reactions with prior UV-deoxygenation (*solid symbols*). Reaction conditions: $[MA]_0/[EBiB]_0/[CuBr_2]_0/[TPMA]_0$ = 100/1/0.04/0.16 in 50% (v/v) DMSO; ultrasound source (40 kHz, 110 W).

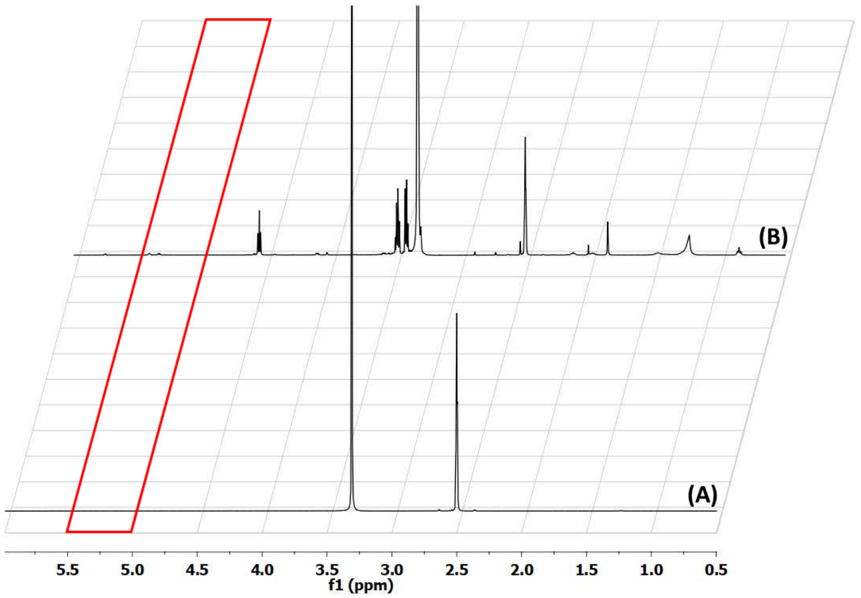

Figure S7. ^{1}H NMR spectra of (**A**) starting DMSO, and (**B**) DMSO supernatant after 4 hours of sonicating dispersion of ZnO (50 mg/mL). The region of expected double bond assignment is denoted.

2.1. Discussion of the electron donor in the absence of excess TPMA for ZnO co-catalyzed photoATRP

Due to the nature of regenerating Cu(I)/L activators from X-Cu(II)/L deactivators, all regenerative-type ATRP mechanisms require an electron donor within the framework. While the ZnO co-catalyst rapidly provides this electron under sono- or photo- stimuli, it ultimately only serves as an intermediate. Subsequently, the electron hole, h^+, residually generated in ZnO should be replenished by an electron donor to cyclically drive the regeneration of Cu activators.

Of the two ZnO co-catalyzed mechanisms investigated in this work, mechanoATRP was found to be simpler with regard to its eligible electron donor species for replenishing electron holes in ZnO. It was found that excess amines (i.e., free excess ligand) readily acted as the sole electron donors to replenish electron holes in ZnO, as in their absence, no polymerization was driven. The donation from amines to electron-deficient ZnO in mechanoATRP has been documented elsewhere.[3]

What was more surprising was that, under analogous photoATRP, excess amines (ligand) were not required to rapidly reach quantitative monomer conversions. While excess amines surely could perform this function, this puzzling result in their absence implies that other species

may serve this function under photoirradiation to quench electron holes – though not under sonication.

For example, electron holes in ZnO generated by UV photoirradiation have been known to be quenched even by water, generating hydroxyl radicals and/or other reactive downstream products that can be scavenged by DMSO.[6] While water was not deliberately added in any way in our work, the polymerizations were not performed under rigorously water-free conditions, as the system was intended to be facile and feasible for non-experts operating under ambient conditions or by simple N_2 sparging.

Regarding solvent, DMSO has not readily been seen to quench electron holes in ZnO nanocrystals made by mechanoATRP, but their potential for contribution under UV photoirradiation is not yet known.[3] Seminal ZnO co-catalyzed ATRP work under UV used acetonitrile, which was not cited as a major electron hole quencher.[7]

Alkyl halide serving as an electron donor would have terrible consequences on the chain-end fidelity and thus molecular weight distributions and initiation efficiencies, as any resulting decomposition would prohibit controlled radical polymerization growth, equivalent to excessive and premature termination of chains. Considering that these consequences were not observed (i.e., a viscous, freely flowing polymer was obtained under controlled conditions), the decomposition of alkyl halide via an electron-transfer pathway to electron holes in ZnO does not appear to be a major pathway. Coincidental supplemental activation by this pathway should be a minor contributor, as our control reaction in the absence of Cu/L showed the formation of an extremely high molecular weight product (**Table S2, entry 1**). It is thus also unlikely that a monomer or polymer contributed as an h^+ quencher through corresponding ester moieties if those of the alkyl halide could not.

One explanation is that the OA capping agent itself, which was not found to act as a reducing agent or quencher alone in the analogous mechanoATRP, may be able to decompose in the presence of UV-generated electron-hole pairs. The process would involve an electron transfer and subsequent decarboxylation at the carboxylic acid moiety. Indeed, just as water is known to quench h^+ to form H^+ and $HO^\cdot$ radicals, it is feasible that the same could occur intra-molecularly with the carboxylic acid moiety of OA to form H^+ and $ROO^\cdot$ radicals, the latter of which would readily decarboxylate.

Furthermore, the selectivity of this process in the photo- variant as opposed to the mechano-variant for quenching by OA may be stimulated by an additional UV photon inducing an excited

state in the ZnO-bound OA, hence the addition of the optional photon (*hv*) in Scheme 2B. This pathway is further rationalized by the intra-molecular nature of this reaction involving OA, as the capping agent is appended directly to the ZnO surfaces. Besides OA, many components could change their redox potentials by entering their excited states via UV excitation, such that the problem becomes quite degenerate. Unfortunately, many other typical capping agents based on amines would fare no better in explaining the problem as they would act as electron donors as well, much in the way excess TPMA ligand would. While the OA capping agent does not ligate to copper, its innocence in quenching electron-deficient ZnO is unknown. Thus, while amines remain the traditional and readily available electron donors for photoexcited (co-)catalysts in ATRP processes, it is beneficial that well-controlled ATRP with OA-capped ZnO co-catalyst could operate without excess amines, likely using residual water and/or (photoexcited) OA for quenching electron holes. Broadly speaking, since any species bearing -OH groups could enter the reaction in this way, it also showcases the capabilities of the photo-variant to accept electrons from a broad family of donors. Since higher performance amine-based ligands like TPMA often form the greatest cost of an ATRP reaction relative to other components, the ability to run this photopolymerization system without the need for their excess relative to Cu offers a cost reduction and improved atom economy.

3. References

(1) Jamatia, T.; Skoda, D.; Urbanek, P.; Sevcik, J.; Maslik, J.; Munster, L.; Kalina, L.; Kuritka, I. Microwave-Assisted Synthesis of FexZn1-xO Nanoparticles for Use in MEH-PPV Nanocomposites and their Application in Polymer Light-Emitting Diodes. *J. Mater. Sci.: Mater. Electron.* **2019**, *30* (12), 11269-11281.

(2) Hammarberg, E.; Prodi-Schwab, A.; Feldmann, C. Microwave-Assisted Polyol Synthesis Of Aluminium- and Indium-Doped ZnO Nanocrystals. *J. Colloid Interface Sci.* **2009**, *334* (1), 29-36.

(3) Wang, Z. H.; Pan, X. C.; Li, L. C.; Fantin, M.; Yan, J. J.; Wang, Z. Y.; Wang, Z. H.; Xia, H. S.; Matyjaszewski, K. Enhancing Mechanically Induced ATRP by Promoting Interfacial Electron Transfer from Piezoelectric Nanoparticles to Cu Catalysts. *Macromolecules* **2017**, *50* (20), 7940-7948.

(4) Ren, Z.; Peng, Y.; He, H.; Ding, C.; Wang, J.; Wang, Z.; Zhang, Z. Piezoelectrically Mediated Reactions: From Catalytic Reactions to Organic Transformations. *Chin. J. Chem.* **2023**, *41*, 111-128.

(5) Wang, Z. H.; Lorandi, F.; Fantin, M.; Wang, Z. Y.; Yan, J. J.; Wang, Z. H.; Xia, H. S.; Matyjaszewski, K. Atom Transfer Radical Polymerization Enabled by Sonochemically Labile Cu-carbonate Species. *ACS Macro Lett.* **2019**, *8* (2), 161-165.

(6) Ong, C. B.; Ng, L. Y.; Mohammad, A. W. A Review of ZnO Nanoparticles as Solar Photocatalysts: Synthesis, Mechanisms and Applications. *Renewable & Sustainable Energy Rev.* **2018**, *81*, 536-551.

(7) Dadashi-Silab, S.; Tasdelen, M. A.; Asiri, A. M.; Khan, S. B.; Yagci, Y. Photoinduced Atom Transfer Radical Polymerization Using Semiconductor Nanoparticles. *Macromol. Rapid Commun.* **2014**, *35* (4), 454-459.
